# A benchmarking study of copy number variation inference methods using single-cell RNA-sequencing data

**DOI:** 10.1093/pcmedi/pbaf011

**Published:** 2025-06-04

**Authors:** Xin Chen, Li Tai Fang, Zhong Chen, Wanqiu Chen, Hongjin Wu, Bin Zhu, Malcolm Moos, Andrew Farmer, Xiaowen Zhang, Wei Xiong, Shusheng Gong, Wendell Jones, Christopher E Mason, Shixiu Wu, Chunlin Xiao, Charles Wang

**Affiliations:** Center for Genomics, School of Medicine, Loma Linda University, Loma Linda, CA 92350, USA; Department of Basic Sciences, School of Medicine, Loma Linda University, Loma Linda, CA 92350, USA; Discovery and Exploratory Statistics, AbbVie Bioresearch Center, Worcester, MA 01605, USA; Bioinformatics Research Engineering, Freenome Holdings Inc., South San Francisco, CA 94080, USA; Center for Genomics, School of Medicine, Loma Linda University, Loma Linda, CA 92350, USA; Department of Basic Sciences, School of Medicine, Loma Linda University, Loma Linda, CA 92350, USA; Center for Genomics, School of Medicine, Loma Linda University, Loma Linda, CA 92350, USA; Department of Basic Sciences, School of Medicine, Loma Linda University, Loma Linda, CA 92350, USA; Center for Genomics, School of Medicine, Loma Linda University, Loma Linda, CA 92350, USA; Division of Cancer Epidemiology and Genetics, National Cancer Institute, National Institutes of Health, 9609 Medical Center Drive, Bethesda, Maryland 20892, USA; Center for Biologics Evaluation and Research, Office of Cellular Therapies and Human Tissues, U.S. Food and Drug Administration, Silver Spring, Maryland 20993, USA; Takara Bio USA, Inc., San Jose, CA 95131, USA; Department of Otolaryngology, the First Affiliated Hospital of Guangzhou Medical University, Guangzhou 510182, China; Department of Otolaryngology, Beijing Friendship Hospital, Capital Medical University, Beijing 100050, China; Department of Otolaryngology, Beijing Friendship Hospital, Capital Medical University, Beijing 100050, China; IQVIA Laboratories Genomics, Durham, NC 27703, USA; Department of Physiology and Biophysics, Weill Cornell Medicine, New York, NY 10065, USA; Quzhou Hospital, Wenzhou Medical University, Quzhou 324000, China; National Center for Biotechnology Information, National Library of Medicine, National Institutes of Health, Bethesda, MD 20894, USA; Center for Genomics, School of Medicine, Loma Linda University, Loma Linda, CA 92350, USA; Department of Basic Sciences, School of Medicine, Loma Linda University, Loma Linda, CA 92350, USA

**Keywords:** scRNA-seq, RNA-seq, copy number variation (CNV) inference, scRNA-seq CNV methods, benchmarking

## Abstract

**Background:**

Single-cell RNA-sequencing (scRNA-seq) has emerged as a powerful tool for cancer research, enabling in-depth characterization of tumor heterogeneity at the single-cell level. Recently, several scRNA-seq copy number variation (scCNV) inference methods have been developed, expanding the application of scRNA-seq to study genetic heterogeneity in cancer using transcriptomic data. However, the fidelity of these methods has not been investigated systematically.

**Methods:**

We benchmarked five commonly used scCNV inference methods: HoneyBADGER, CopyKAT, CaSpER, inferCNV, and sciCNV. We evaluated their performance across four different scRNA-seq platforms using data from our previous multicenter study. We evaluated scCNV performance further using scRNA-seq datasets derived from mixed samples consisting of five human lung adenocarcinoma cell lines and also sequenced tissues from a small cell lung cancer patient and used the data to validate our findings with a clinical scRNA-seq dataset.

**Results:**

We found that the sensitivity and specificity of the five scCNV inference methods varied, depending on the selection of reference data, sequencing depth, and read length. CopyKAT and CaSpER outperformed other methods overall, while inferCNV, sciCNV, and CopyKAT performed better than other methods in subclone identification. We found that batch effects significantly affected the performance of subclone identification in mixed datasets in most methods we tested.

**Conclusion:**

Our benchmarking study revealed the strengths and weaknesses of each of these scCNV inference methods and provided guidance for selecting the optimal CNV inference method using scRNA-seq data.

## Introduction

Genetic heterogeneity, both inter-tumor and intra-tumor, has been reported extensively in various cancer types [1–6]. Copy number variation (CNV) plays a crucial role in cancer development and progression by amplifying oncogenes or inactivating tumor suppressor genes [7–10]. CNV is an important parameter for characterizing inter-tumor and intra-tumor heterogeneity. While recent studies have highlighted the impact of somatic CNVs on gene expression levels in bulk cell analyses [11–13], the relationship between single-cell genetic and transcriptional heterogeneity remains unclear. The rapid development of single-cell RNA and DNA sequencing technologies [14–18] has allowed researchers to study this question directly. However, simultaneous assessment of RNA and DNA information from the same cell remains technically challenging, limiting studies [19, 20] that integrate subpopulations characterized by transcript abundance and genetic alterations at single-cell resolution.

To address this challenge, several methods have been developed recently to infer CNVs from single-cell RNA sequencing (scRNA-seq) data: HoneyBADGER [21], inferCNV [22], sciCNV [23], CaSpER [24], and CopyKAT [25]. These methods enable the integrated analysis of RNA and DNA at the single-cell level. HoneyBADGER [21] utilizes a hidden Markov model (HMM) integrated with a Bayesian method for CNV detection, providing both expression-based and allele-based CNV inference. Like the expression-based method, HoneyBADGER [21], inferCNV [22] implements a similar strategy for CNV detection but uses different data de-noising, smoothing, and normalization strategies and considers more CNV states in a transition matrix for CNV detection. sciCNV [23] calculates an expression disparity score combined with another score that assesses concordant expression changes in contiguous genes to identify CNV. CaSpER [24] adopts a signal processing approach, performing multiscale smoothing of both gene expression and allele frequency, integrating these two types of data for CNV detection. CopyKAT [25] uses a statistical model to identify and characterize cellular subpopulations, while considering technical noise and biological variation in gene expression for assessing cell-to-cell variation. All five methods claim to be able to detect both large-scale and focal CNVs. However, these methods have not been evaluated comprehensively with scRNA-seq data derived from different platforms under different sequencing settings and clinical applications.

Here we carried out a comprehensive study to assess the effects of scRNA-seq platform, sequencing depth, and bioinformatics algorithm on scRNA-seq CNV analysis. First, we assessed the sensitivity and specificity of the five CNV inference methods using scRNA-seq data derived from a multicenter benchmarking study on the cell lines (HCC1395/HCC1395BL) based on different scRNA-seq platforms and sequencing depths. We evaluated various factors including cell numbers, read lengths, read depths, and reference data for the four scRNA-seq platforms. Second, we evaluated the accuracy of tumor subpopulation identification of each scCNV inference method using a scRNA-seq dataset derived from mixed samples consisting of five human lung adenocarcinoma cell lines. The accuracy was assessed by comparing estimated tumor subpopulations and known cell lines using metrics such as Adjusted Rand Index (ARI) [26], Fowlkes-Mallows index (FM) [27], Normalized Mutual Information (NMI) [28], and V-Measure [29]. Finally, we generated a clinical dataset to validate our findings. Overall, we found that the five methods exhibited large discordance. CaSpER and CopyKAT showed better performance in terms of sensitivity and specificity in CNV inference using scRNA-seq data, whereas inferCNV and CopyKAT exhibited the best performance for identifying tumor subpopulations in the data derived from a single scRNA-seq platform. However, the expression-based CNV inference methods such as inferCNV, CaSpER, sciCNV, and CopyKAT were highly affected by batch effects (e.g., scRNA-seq platform) when estimating tumor subpopulations using datasets derived from multiple platforms. Our evaluation, based on the clinical dataset, showed that CaSpER and CopyKAT outperformed other methods in terms of sensitivity and specificity of CNV inference. However, regarding subpopulation identification, inferCNV and CopyKAT achieved more accurate results. Our findings revealed the limitations of the current CNV inference methods using scRNA-seq data and provided some guidelines for developing new ones.

## Methods

### Datasets used in our study

We used three different data sets, including scRNA-seq data from a multicenter benchmark study [30], scRNA-seq data from mixed human lung adenocarcinoma cell line samples [31], and the newly generated clinical human small cell lung cancer data. These datasets were as follows.

### scRNA-seq datasets from a multicenter study

We used the scRNA-seq datasets from our previous multi-center benchmarking study, which were obtained from a human breast cancer cell line (HCC1395) vs. the matched “normal” control B lymphocyte cell line (HCC1395BL) from the same donor [30, 32–35]. The scRNA-seq datasets were generated from four scRNA-seq platforms including two full-length transcript techniques: Fluidigm C1 (referred to as C1) and Takara Bio's ICELL8 (referred to as ICELL8), and two tag-based 3’-transcript techniques: 10x Genomics (hereafter referred to as 10x) and Fluidigm C1 HT (hereafter referred to as C1 HT). Detailed methods describing how the scRNA-seq data were generated were described in our previous publication [30]. The multi-center scRNA-seq fastq raw data is available in the SRA repository with the access code #: PRJNA504037.

### scRNA-seq data from mixed human lung adenocarcinoma cell line samples

We also used the Tian et al. scRNA-seq dataset [31], derived from mixed samples consisting of either three or five human lung adenocarcinoma cell lines, to mimic tumor subpopulations [31]. These datasets were generated using three tag-based techniques: 10x, CEL-seq2, and Drop-seq. The detailed methods on how these mixed scRNA-seq data were generated can be found in the Tian et al paper [31]; these data are available under GEO SuperSeries GSE118767.

### Clinical human small cell lung cancer data

***Tissue collection:*** All sample collection, patient consent, and patient recruitment followed Institutional Review Board (IRB) platforms approved by the Hangzhou Cancer Hospital, Hangzhou, China. The samples were obtained from a 59-year-old male with an initial diagnosis of left lung cancer by a chest computed tomography (CT) scan. The patient underwent further CT-guided lung biopsy yielding three pieces of cancer tissue (0.2×1.5 cm/each) for pathology examination with hematoxylin and eosin (HE) staining, which confirmed a diagnosis of stage 4 small cell lung cancer (SCLC) with liver and bone metastasis. The patient then received chemotherapy. A 10 ml blood sample was obtained as control. One cancer tissue sample was stored at -80 °C, and the other two tissue samples were used immediately for single-cell isolations. When the patient relapsed after chemotherapy, two pieces of cancer tissue (0.2×1.5 cm/each) were obtained by CT-guided lung puncture. One sample was stored at -80 °C, the other was used for single-cell isolations. For the tissue collections both during the primary and relapse periods, adjacent normal tissues (3 perforated tissues) were also obtained from the patient; these were also subject to HE staining and pathology confirmation as peritumoral normal tissues.

***Bulk cell WGS:*** genomic DNA (gDNA) was extracted from the fresh frozen tissues using the Qiagen QIAamp DNA mini kit (Qiagen, Germany) according to manufacturer's instructions. The DNA sample quality and integrity were evaluated by A260/A280 ratio and agarose gel electrophoresis. The concentration of gDNA was determined by Nanodrop 2000 (Thermo, USA) and Qubit 3.0 (Life Technologies, USA). WGS libraries were constructed using the Illumina Tru-seq Nano DNA HT Sample Prep kit with 0.5 µg gDNA following the manufacturer's procedure and sequenced on an Illumina X10, paired end (PE, 150 bpx2) with 30x coverage. The fastq sequencing data were deposited in the SRA database, under SRA number SRP149859.

***Single cell isolation:*** The tissues were digested with collagenase IV (Sigma Aldrich, Shanghai, China), at 37 °C for 30 min, washed in phosphate-buffered saline, and then diluted to 1000 cells/ml. Single cells were isolated using a micromanipulation system [36].

***Single-cell RNA-seq (scRNA-seq):*** cDNAs were amplified from 92 primary tumor cells and 39 relapse tumor cells using the SMART-seq2 kit as described previously [37]. scRNA-seq libraries were constructed with 1 ng amplified cDNA using the Nextera XT DNA Library Preparation kit (Illumina, San Diego, CA, USA) following the manufacturer's protocol. The scRNA-seq libraries were sequenced with 20M reads per cell on an Illumina X10 with PE reads, 150 pbx2. The fastq sequencing data were deposited in the SRA database, under SRA number SRP149859.

***Single-cell WES (scWES):*** Single-cell genomic DNA obtained from 69 primary tumor cells and 76 relapse tumor cells were amplified with the Qiagen REPLI-g Single Cell kit (Qiagen, Shanghai, China) as described previously [36]. Exome capture was performed on amplified DNA from each single cell using the Agilent SureSelect Clinical Research Exome panel (Agilent Technologies, Shanghai, China) following the manufacturer's instructions. The Agilent SureSelect Clinical Research Exome panel targets a 54 Mb region including gene exons. The captured DNAs were purified using AMPure XP beads (Beckman Coulter). The scWES libraries were sequenced on an Illumina X10 with PE reads, 150 bpx2, with 200x coverage for each cell. The fastq sequencing data were deposited in the SRA database, under SRA number SRP152993.

### Human reference genome

The reference genome and transcriptome were downloaded from the 10X website as refdata-cellranger-GRCh38-1.2.0.tar.gz. This reference corresponds to the GRCh38 genome and Ensmebl v84 transcriptome. All data analyses were performed using this reference genome and transcriptome.

### Preprocessing of scRNA-seq data

For 10x data, the raw fastq data were processed using CellRanger (v2.1.0 and v3.1.0) to generate gene count matrices. In the CellRanger pipeline, cellranger count was used with all default parameter settings.

For C1, C1-HT, and ICELL8 data, the quality of the raw fastq data was assessed by fastqc. cutadapt (v1.9.1) was used for trimming and filtering. Bases with quality less than 10 were trimmed from 5’ and 3’ ends of reads. Reads less than 20 bases were excluded from further analysis. STAR (v2.5.4b) with default parameter settings was used for alignment to generate bam files. featureCounts (v1.6.1) was used to generate gene counts per cell with all default parameter settings.

For Tian's Drop-seq and CEL-seq2 data, umitools (v1.0.0) was used to process the raw fastq data and generate gene count matrices. In the umitools pipeline, ‘umi_tools whitelist’ with default parameter settings was used to generate a list of cell barcodes for downstream analysis. ‘umi_tools extract’ was used to extract the cell barcodes and filter the reads if the phred sequence quality of the cell barcode bases was < 10 or UMI bases < 10 (options: –quality-filter-threshold=10 –filter-cell-barcode). STAR was used for alignment to generate bam files containing the unique mapped reads (option: outFilterMultimapNmax 1). For gene counting, featureCounts was used to assign reads to genes and generate a BAM file (option: -R BAM). Finally, ‘umitools count’ (options: –per-gene –gene-tag=XT –per-cell –wide-format-cell-counts) was used for the sorted BAM files to generate gene count per cell matrices.

### CNV inference methods

Heterozygous SNP identification for HoneyBADGER: Three heterozygous SNP reference sets were used in the HoneyBADGER allele-based method. For SNPs sourced from ExAC, common heterozygous variants in Variant Call Format (VCF) were extracted from the database. These variants underwent filtering to include only common SNPs with a minor allele frequency (MAF) greater than 10%.

Consensus Single Nucleotide Polymorphisms (SNPs) identified from 63 HCC1395BL WGS samples followed the data processing and VCF extraction guidelines outlined in a reference paper [34]. The same variant filtering strategy was applied to obtain the final SNP references.

In one WES sample, the fastq files were trimmed using cutadapt (v1.9.1). Reads less than 20 bases were excluded from further analysis. The trimmed fastq files were aligned to the human reference genome (GRCh38) using BWA MEM (v0.7.13). Duplicates were removed using Picard tools (v1.141). Indel realignment and quality score recalibration were performed using GATK v3.8.1. Germline heterozygous variants were identified using GATK's HaplotypeCaller, followed by variant quality score recalibration. The same variant filtering strategy was applied to obtain the final SNP reference set.

HoneyBADGER: For expression-based analysis (HoneyBADGER-expression), the log-transformed counts per million (CPM) were used as input, and the following gene filtering parameters were applied: Genes with mean expression lower than 1 CPM in both test and control samples, genes with mean expression lower than 3.5 CPM in the test sample, or genes with mean expression lower than 2.6 CPM in the control sample were filtered from further analysis.

For the allele-based analysis (HoneyBADGER-allele), SNPs with greater than 0.05 deviation from the expected 0.5 heterozygous allele fraction were excluded from for further analysis.

InferCNV: The inferCNV analysis involved the following steps: “denoise” was performed using the default hidden Markov model (HMM) settings, with a “cutoff” value of 0.1 for tag-based platforms and 1 for full-length platforms. The “subcluster” method was applied to infer the subcluster cells using the “random_trees” partition method. The default p-value of 0.05 was used to determine cut-points in the hierarchical tree.

CaSpER: BAFExtract was used to obtain BAF for each bam file of the scRNA-seq data. The log-transformed counts per million (CPM) were used as gene expression matrices. The CaSpER analysis was performed in “single-cell” mode, with expressed genes filtered out when their expression was less than 0.1.

sciCNV: The gene count matrices were normalized using the RTAM2 normalization method developed by the same group. The sciCNV analysis was performed on the normalized gene expression matrices with default parameter settings. The sciCNV scores per gene per cell were calculated for further analysis.

CopyKAT: CopyKAT analysis was performed using default parameter settings.

### Cytoband-based CNV comparison

In the cytoband-based CNV comparison, we started by defining the ground-truth CNVs based on their cytoband locations. This allowed us to generate total events, positive events, and negative events, and further calculate sensitivity and specificity. We considered CNVs reported from the two reference papers [7, 38] as total events. To determine the positive events, we identified the CNVs that overlapped between the total events and the CNVs identified in the WGS data using subHMM. The CNVs that did not overlap were considered negative events.

Due to the variations in CNV output among the five scCNV inference methods, we converted the CNV output into gene-based CNVs for downstream analysis. This involved assigning CNVs to genes at the single cell level if they overlapped with the genomic region of the CNVs. Subsequently, we defined consensus gene-based CNVs if the genes were called in more than 10% of the cells. Using the cytoband location of gene-based CNVs and the ground truth, we calculated sensitivity and specificity. ROC curves were generated based on the consensus gene-based CNVs with different cell percentage cutoffs ranging from 5% to 100%.

### Cell type identification for Tian's data

To determine the cell types identified in Tian's scRNA-seq data, we initially obtained bulk RNA-seq data from the five lung cancer cell lines (GSE64098) [39]. We performed a differential gene expression analysis on this bulk RNA-seq data to identify the top 5 gene marks for each of the five cell lines.

For scRNA-seq datasets, we applied the standard preprocessing and clustering steps using the Seurat package. The identified gene marks for each of the cell lines from the bulk RNA-seq data were used as inputs to generate feature plots to determine the cell lines for estimated clusters.

### Metric score calculation

To calculate the four metric scores (ARI, FM, NMI, and V-Measure), we used several R packages: fossil (v0.4.0), dendextend (v1.13.4), aricode (v1.0.0), and infotheo (v1.2.0). Each of these packages was used for specific calculations related to the four metrics. To calculate the scores, hierarchical clustering was performed on the outputs of the five CNV inference methods. Then the metric scores were calculated based on the cluster labels in hierarchical clustering and the true cell type labels or batch labels. Here is a description of the hierarchical clustering approach used for each CNV inference method:

- HoneyBADGER: hierarchical clustering was applied to the posterior probability of each CNV in each cell.
- inferCNV: tumor subclustering was used to obtain hierarchical clustering results.
- CaSpER: hierarchical clustering was applied to smoothed genome-wide gene expression data.
- sciCNV: hierarchical clustering was applied to sciCNV scores derived from the method.
- CopyKAT: the default hierarchical clustering provided by the CopyKAT method was used.

### Metrics to evaluate rare subpopulation identification

To evaluate the performance of the five scCNV inference methods in identifying rare subpopulations, we employed three metrics: percentage of successful runs, number of clusters required to identify the rare cell subpopulation (referred to as number of clusters), and the proportion of cells labeled as rare subpopulation cells (referred to as cell proportion).

We used hierarchical clustering to estimate clusters. We constructed a contingency table comparing the estimated cluster labels with the cell line labels. Initially, we set the estimated number of clusters to three and gradually increased the number of clusters until we obtained one cluster that included only the rare subpopulation cells. The upper limit of the number of clusters was set at 30. If the number of clusters exceeded 30, the run was considered a failure.

To ensure robustness, we performed 10 runs for the same dataset using each scCNV inference method, considering the HMM approach employed in these methods. The percentage of successful runs represented the number of successful runs out of 10 repeated runs for each dataset and scCNV method. The cell proportion metrics indicated the proportion of rare subclonal cells within the estimated cluster compared with the total number of rare subclonal cells.

## Data availability

The datasets generated and analyzed (multi-center scRNA-seq data) in the current study are available in the SRA repository with the access code #: PRJNA504037, and the following URL: https://www.ncbi.nlm.nih.gov/bioproject/PRJNA504037

The raw data from the Tian et al. mixture scRNA-seq data for the five lung cancer cell lines are available under GEO SuperSeries GSE118767, and the following URL: https://www.ncbi.nlm.nih.gov/geo/query/acc.cgi?acc=GSE118767

The raw data for human SCLC is available in the SRA repository with the accession code #: SRP152993 (scWES and bulk cell WGS), SRP149859 (scRNA-seq) and the following URL: https://trace.ncbi.nlm.nih.gov/Traces/?view=study&acc=SRP15299

https://trace.ncbi.nlm.nih.gov/Traces/?view=study&acc=SRP149859

## Code availability

The algorithms and code for our bioinformatics analyses have been published previously (https://github.com/xchen004/CNV_inference_benchmark).

## Results

### Overall study design and associated datasets

**Figure 1.**
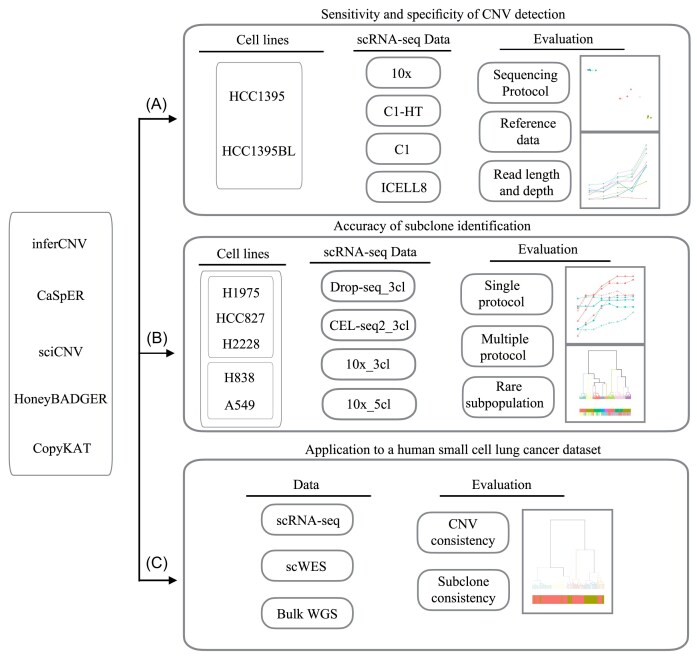
Study design of the scCNV benchmark analysis. The top panel **(A)** illustrates the evaluation scheme for sensitivity and specificity of scCNV detection using the scRNA-seq datasets (10x, C1-HT, C1, and ICELL8 full-length) of a breast cancer cell line vs. the paired B-cell line derived from the same donor, which was generated from our previous multicenter benchmarking study. The middle panel **(B)** illustrates the evaluation scheme for accuracy of subclone identification using the mixed scRNA-seq data from the Tian et al. study derived from a mixture including either three or five human lung adenocarcinoma cell lines. Drop-seq_3cl, scRNA-seq data from the mixed three human lung adenocarcinoma cell lines; CEL-seq2_3cl, scRNA-seq data from the mixed three human lung adenocarcinoma cell lines; 10x_3cl, 10x scRNA-seq data from the mixed three human lung adenocarcinoma cell lines; and 10x_5cl, 10x scRNA-seq data from the mixed five human lung adenocarcinoma cell lines. The lower panel **(C)** illustrates the application of scCNV methods to a human small cell lung cancer (SCLC) scRNA-seq dataset (20M read/each cell, full-length transcript, SMART-seq2) including 92 primary SCLC single cells and 39 relapse SCLC single cells, plus scWES and bulk cell WGS from primary SCLC and relapsed tumoral tissues as well as peri-tumoral normal tissues.

To evaluate the sensitivity and specificity of the CNV inference methods, we utilized scRNA-seq data derived from our previous multi-center benchmarking study, which used paired tumor/normal samples from a human breast cancer cell line (HCC1395, referred to as Sample A) and a matched “normal” control cell line derived from B lymphocytes (HCC1395BL, referred to as B) from the same donor [30, 32–35]. Four scRNA-seq platforms were employed [30], including two full-length transcript techniques: Fluidigm C1 (referred to as C1) and Takara Bio's ICELL8 (referred to as ICELL8), as well as two tag-based 3’-transcript techniques: 10x Genomics (hereafter referred to as 10x) and Fluidigm C1 HT (hereafter referred to as C1 HT) (Fig. 1A). In our study, we first determined the CNVs for the breast cancer cell line based on the bulk cell whole-genome sequencing (WGS) and we detected numerous CNVs [34, 35]. However, to increase the stringency and to make a conservative choice about which of these are most likely true positive CNV events, we chose only the 79 CNVs which were also reported by Zack et al. [7] to be highly recurrent in breast cancers (e.g., the amplification of the MYC oncogene at 8q24.21) as ground truth (based on biology with high stringency) for our evaluation study. Of these 79 CNVs (34 gains and 45 losses), 26 CNVs (11 gains and 15 losses), which were identified in our breast cancer cell line using subHMM [40] based on bulk cell WGS, were regarded as true positive events, and the remaining 53 CNVs were considered negative events.

To examine the accuracy of tumor subpopulation identification, we used the Tian et al. scRNA-seq dataset [31], derived from samples consisting of mixtures of either three or five human lung adenocarcinoma cell lines to mimic tumor subpopulations [31]. These datasets were generated using three tag-based techniques: 10x, CEL-seq2, and Drop-seq (Fig. 1B).

To evaluate the clinical relevance and to validate our results based on the above cell line data, we generated a clinical dataset derived from a human small cell lung cancer (SCLC) which is comprised of scRNA-seq data from 92 primary SCLC single cells (20M reads/cell, full-length transcript, SMART-seq2) and 39 relapse SCLC single cells (20M reads/cell, full-length transcript, SMART-seq2), as well as single-cell whole-exome sequencing (scWES, 200x coverage/cell) from 69 primary SCLC single cells, 76 relapse SCLC single cells, and 10 peri-tumoral normal lung single cells plus bulk cell whole-genome sequencing (WGS, 60x coverage/each) data from the primary tumor tissue, relapse tumor tissue, and peritumoral tissue (Fig. 1C).

### Sensitivity and specificity of CNV inference by scCNV methods

Cytoband-based CNV comparison was used for the evaluation (see “Methods”). Briefly, 79 highly recurrent CNVs [7], as described above, were considered as total events. These included 34 CNV gains and 45 losses that were denoted by their cytoband locations. CNVs (at cytoband levels) identified by subHMM [40] in the WGS data were used as ground truth to assess sensitivity and specificity of scCNV callers using scRNA-seq data. Among these 79 highly recurrent CNVs, 26 CNVs (11 CNV gains and 15 CNV losses) identified in the WGS data were considered positive events, while the remaining 53 CNVs were considered negative events. In this study, five scCNV methods, inferCNV, CaSpER, CopyKAT, sciCNV, and HoneyBADGER (both expression- and allele-based) were applied to scRNA-seq datasets derived from four different scRNA-seq platforms (Fig. 1A) to identify CNVs and further annotated at cytoband levels (see “Methods”). In addition, we also applied these five scCNV methods to the bulk RNA-seq data (as a control for evaluation) but found that only CaSpER and CopyKAT could identify CNVs successfully. We included the results of bulk RNA-seq as a control for these two methods in the evaluation.

**Figure 2.**
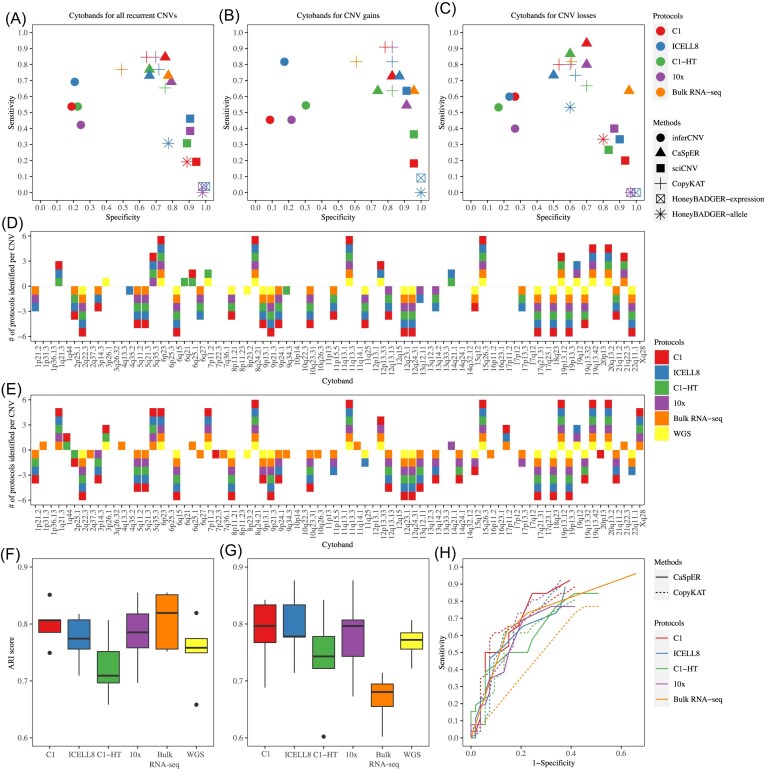
Sensitivity, specificity, and consensus evaluation of scCNV inference methods. (**A-C**) Sensitivity and specificity of scCNV inference methods applied to four different scRNA-seq plus bulk-cell RNA-seq data sets, considering (**A**) all 79 CNVs, (**B**) CNV gains, and (**C**) CNV losses. (**D**) CaSpER and (**E**) CopyKAT showing the CNV identifications based on 79 highly recurrent CNVs detected using data derived from four different scRNA-seq platforms along with the corresponding bulk-cell RNA-seq and bulk-cell WGS datasets. (**F-G**) Consensus CNVs identified between any two platforms by (**F**) CaSpER and (**G**) CopyKAT. Y-axis refers to the ARI. Each box represents the ARI between CNV identification status of the x-axis labeled platform and other platforms. (**H**) ROC curves of CaSpER and CopyKAT applied to four different scRNA-seq platforms along with bulk-cell RNA-seq dataset.

Overall, CaSpER and CopyKAT showed better performance than the other scCNV methods (Fig. 2A). These two methods did not show significant performance differences across the four scRNA-seq platforms. To compare the performance of scRNA-seq data derived from four different platforms in more detail, we generated Receiver Operating Characteristic (ROC) curves based on the consensus CNVs identified using different cell percentages (see “Methods”, Fig. 2H). The results are consistent with the above findings, with CaSpER performing slightly better in Fluidigm C1 data, while both methods exhibited less favorable performance with the Fluidigm C1-HT data. We extended the evaluation to CNV gains and losses separately (Fig. 2B, C) and observed differences in performance between CaSpER and CopyKAT. In CNV gains, CopyKAT significantly increased sensitivity but slightly sacrificed specificity compared to CaSpER, except with the Fluidigm C1-HT data (Fig. 2B). In CNV loss, the performance of the two methods was scRNA-seq data platform dependent. CaSpER outperformed CopyKAT in both sensitivity and specificity with scRNA-seq data derived from Fluidigm C1 and 10x platforms, whereas CopyKAT showed better specificity with the ICELL8 scRNA-seq data (Fig. 2C). In addition, CopyKAT performed better at detecting CNV gains than CNV losses. It outperformed in sensitivity and specificity, or both with the four different platform derived scRNA-seq data sets (Fig. 2B, C). The staggered bar charts (Fig. 2D, E) illustrate the consistency of the 79 highly recurrent CNVs identified across the five different data sets, including the bulk RNA-seq data, for CaSpER and CopyKAT, respectively.

In Figure 2D and E, consensus CNVs were defined as those identified in all five datasets, while low-concordance CNVs were identified in two or fewer platform derived data sets. CopyKAT detected more low-concordance CNVs than CaSpER in both CNV gains (9 vs. 6) and CNV losses (12 vs. 4). Most of these low-concordance CNVs (5 in CNV gains, 7 in CNV losses) originated from the bulk RNA-seq data, indicating a larger number of falsely identified CNVs, which contributed to the lower specificity compared with CaSpER (Fig. 2B, C). In terms of consensus CNVs, CopyKAT detected more CNV gains (9 vs 4), but the number was comparable for CNV losses (14 vs 13) compared with CaSpER, resulting in higher sensitivity for CNV gains across the three scRNA-seq data (C1, 10x, and ICELL8).

To assess the consistency of scCNV identification across data derived from different scRNA-seq platforms, we measured the similarity of CNV detection status using the ARI. Box plots of ARI between a selected platform derived data and any one of the other platform derived data were generated for CaSpER (Fig. 2F) and CopyKAT (Fig. 2G). The bulk RNA-seq data were also included as a control for ARI analysis. The Fluidigm C1-HT and bulk RNA-seq showed the lowest consistency for CaSpER and CopyKAT (Fig. 2F, G, Supplementary Fig. 1A-D), suggesting that these methods may not perform optimally for Fluidigm C1-HT and bulk RNA-seq data. The ROC curves (Fig. 2H) confirmed the subpar performance of these two methods for Fluidigm C1-HT and bulk RNA-seq data. We also evaluated the consistency between any two platforms for CNV gains and losses. In CaSpER, no significant difference was observed between CNV gains and CNV losses (Supplementary Fig. 1E). In CopyKAT, the ARI score was higher for CNV gains (Supplementary Fig. 1F), indicating better performance.

### Effect of scRNA-seq CNV detection using different RNA-seq reference datasets

Reference data are required as controls to perform scCNV inference for CNV calling. In our evaluation, we examined three different reference datasets using the four scCNV inference methods, excluding HoneyBADGER due to its extremely low sensitivity.

**Figure 3.**
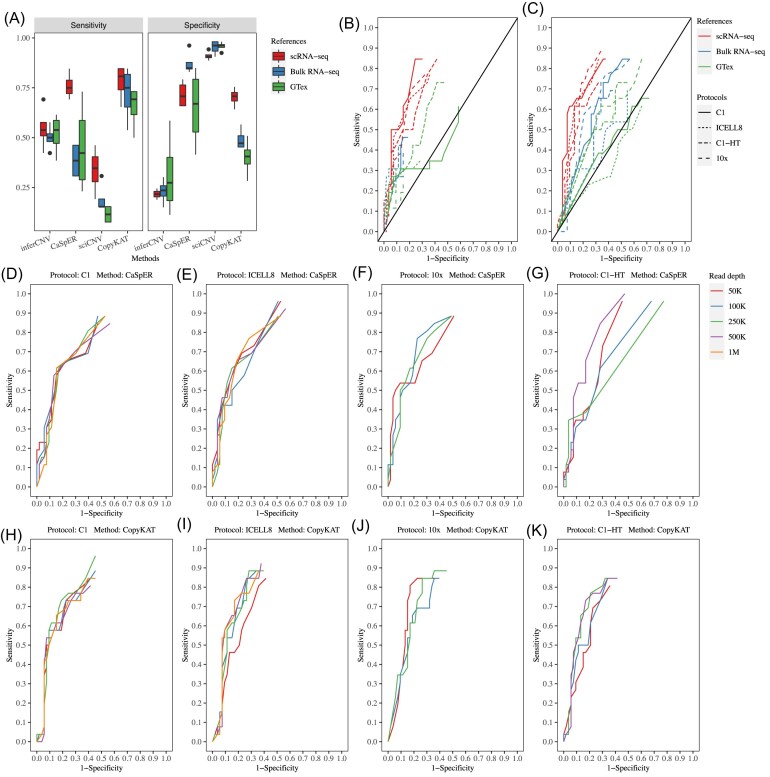
Sensitivity and specificity of scCNV inference methods under multiple conditions. **(A)** Sensitivity and specificity of the four scCNV inference methods using three reference datasets. Each box represents the sensitivity or specificity of the x-axis labeled method applied to four scRNA-seq platforms. **(B-C)** ROC curves of CaSpER (**B**) and CopyKAT (**C**) applied to four scRNA-seq platforms using three different reference datasets. (**D-K**) ROC curves of CaSpER (**D-G**) and CopyKAT (**H-K**) applied to four scRNA-seq platforms. The reads of each scRNA-seq data set were down sampled to specific read depths.

For this evaluation, we used the following three reference datasets: scRNA-seq data from sample B (HCC1395BL), bulk RNA-seq data from sample B (HCC1395BL), and bulk RNA-seq data from normal breast tissue in the GTex database [41]. Twelve cases in total were evaluated for each scCNV method when three reference datasets and four scRNA-seq platforms were considered. In general, sensitivity decreased significantly in all scCNV inference methods (except inferCNV) across four scRNA-seq platforms when the two bulk RNA-seq reference data sets were used (Fig. 3A). However, when using bulk RNA-seq data from the GTex database, most scCNV methods showed lower sensitivity and/or specificity (Fig. 3A). The findings highlight the importance of selecting an appropriate reference dataset. Ideally, a matched scRNA-seq dataset from normal cells is preferred. If scRNA-seq data are unavailable, a matched bulk RNA-seq dataset can serve as the next best alternative.

We analyzed the performance of CaSpER and CopyKAT with the three reference datasets in more detail since the two methods showed the better balance between sensitivity and specificity. The ROC curves of CaSpER (Fig. 3B) and CopyKAT (Fig. 3C) suggested that both methods performed better with scRNA-seq reference data than with bulk RNA-seq reference data. However, when using the two bulk RNA-seq reference datasets (Fig. 3B-C), CaSpER failed to call concordant CNVs with high cell percentages (Supplementary Fig. 2), resulting in the apparent low sensitivity in the ROC curves in 6 out of 8 cases. Of these six scenarios, more than 57% of CNVs could be called in fewer than 5% of cells. This resulted in only a limited number of concordant CNVs being called in a high percentage of cells (Supplementary Table 1). In contrast, when using CopyKAT, fewer than 21% of CNVs were called in less than 5% of the cells in the 8 scenarios using the same RNA-seq reference datasets (Supplementary Table 1).”

### The effect of read length and read depth of scRNA-seq on scCNV inferences

To assess the impact of read length and read depth, we focused on CaSpER and CopyKAT. We evaluated five different read lengths (50bp, 75bp, 100bp, 125bp, and 150bp) and read depths (50k, 100k, 250k, 500k, and 1M). For CopyKAT, the ROC curves did not exhibit noticeable differences across five read lengths and five read depths (Fig. 3H-K, Supplementary Fig. 4). Conversely, for CaSpER, the ROC curves were affected substantially by both read length and depth, except for the Fluidigm C1 platform (Fig. 3D-G, Supplementary Fig. 3). The AUCs corresponding to the iCELL8, C1-HT, and 10x platforms showed drastic decreases at specific combinations of read length and read depth (Fig. 3G, Supplementary Fig. 3F-I, K, M, N, Q). However, the ROC curves were not monotonically related to the read depth or read length. Further analysis revealed that the unstable performance was due to use of different B-allele frequencies (BAFs) files at different read lengths and depths, leading to remarkably different BAF shift signal profiles to call CNVs.

To validate this presumption, we used 100bp SE data from the ICELL8 platform (Supplementary Fig. 3H). The initial analysis (Supplementary Fig. 3M) showed high and low AUCs at read depths of 500K and 1M, respectively. We repeated CaSpER analysis on 100bp SE data at five different read depths, but instead of using the BAF files corresponding to each read depth, we replaced them with the BAF files from the read depths at 500K or 1M, separately (Supplementary Fig. 5A). For simplicity, we named these two BAF files as 500K-BAF and 1M-BAF. Consistently low AUCs were observed across the five read depths when the 1M-BAF file was used. In contrast, the AUCs increased when the 500K-BAF file was used (Supplementary Fig. 5A), indicating that the performance of CaSpER was affected by the BAF file. Similar results were obtained when extending the analysis to other cases in ICELL8 (125bp SE: 500K-BAF vs 250K-BAF) and Fluidigm C1-HT (100bp SE: 250K-BAF vs 500K-BAF; 125bp SE: 500K-BAF vs 250K-BAF) platforms (Supplementary Fig. 5B-D). Overall, CaSpER was indeed affected by both read length and read depth when using different BAF files.

### Accuracy of scCNV inferred subpopulations using mixed scRNA-seq lung cancer cell line datasets

One of the major applications of scRNA-seq CNV inference methods is the identification of tumor subpopulations. To mimic these subpopulations, we used a mixed sample scRNA-seq dataset obtained from public database GSE118767 [31], which consists of four datasets with mixtures of either three (H1975, H2228, HCC827) or five (A549, H1975, H2228, H838, HCC827) human lung adenocarcinoma cancer cell lines. The mixed scRNA-seq datasets were generated using three different platforms: Drop-seq, CEL-Seq2, or 10x Genomics. We referred to the datasets as Drop-seq_3cl, CEL-seq2_3cl, 10x_3cl, and 10x_5cl, respectively. In our evaluation, we considered three or five cancer cell lines as the major subpopulations, making the following assumptions: (1) Each cell line comprises relatively homogeneous clonal cells; and (2) Different cell lines exhibit high heterogeneity. We assessed the performance of the scCNV inference methods using hierarchical clustering trees, ARI, FM, NMI, V-Measure, and number of estimated clusters. We considered three evaluation scenarios for subpopulation identification based on: a single scRNA-seq platform; combined multiple scRNA-seq platforms; or rare subpopulation inference.

#### Subpopulation inference based on data from a single scRNA-seq platform

**Figure 4.**
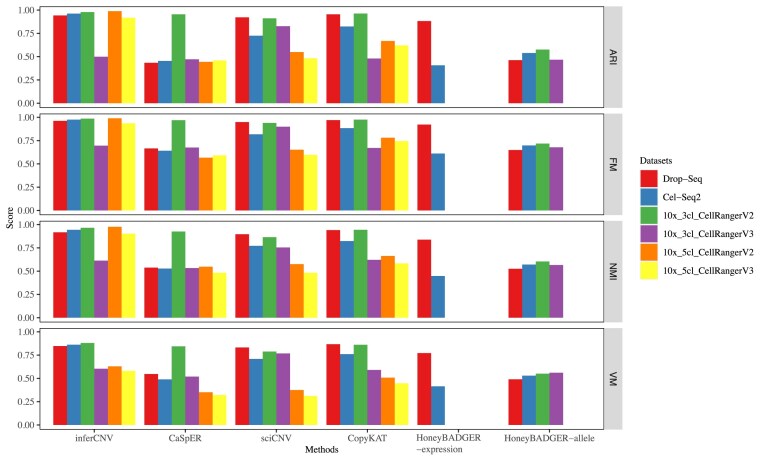
Accuracy of subpopulation identification based on scRNA-seq data from a single platform. Four measurement scores, Adjusted Rand Index (ARI), Fowlkes-Mallows index (FM), Normalized Mutual Information (NMI), and V-Measure were used to evaluate the accuracy in six datasets. The measurement scores were calculated between the estimated clusters and the true cell type labels. The number of clusters estimated was identical to the number of true cell types.

In this scenario, we evaluated the scCNV inference methods individually on the four datasets. We processed the Drop-seq and CEL-seq2 datasets using umitools (v1.0.0), while both Cell Ranger V2 and V3 were used to process 10x_3cl and 10x_5cl datasets. This resulted in a total of six gene count matrices (referred to as datasets) for evaluation. Methods that achieved higher ARI, FM, and V-Measure scores were considered to have better performance. HoneyBADGER-expression (expression-based method) could not identify CNVs in any of the 10x datasets, so it was not considered further in the evaluation. Similarly, HoneyBADGER-allele (allele-based method) was not considered further in the evaluation for 10x_5cl datasets. We found that inferCNV, sciCNV, and CopyKAT performed better than CaSpER or HoneyBADGER (Fig. 4). inferCNV outperformed sciCNV and CopyKAT in the 10x_5cl datasets, suggesting its ability to identify cell subpopulations in more complex and heterogeneous scenarios (Fig. 4). Another notable finding was that sciCNV was less affected by 10x_3cl datasets processed by the two Cell Ranger pipelines (Fig. 4, Supplementary Fig. 6C, D). The metrics scores of the two 10x_3cl datasets were similar in sciCNV but not in inferCNV or CopyKAT (Fig. 4). Interestingly, we observed that most of the additional low RNA content cells called by Cell Ranger V3 were HCC827 cells (631 out of 636 cells). In the case of inferCNV and CopyKAT, hierarchical clustering failed to merge the HCC827 cells called by Cell Ranger V2 and V3 (Supplementary Fig. 6A, B, E, F). Instead, these methods treated the low RNA content HCC827 cells as a unique subpopulation. To assess the ambient RNA contamination levels in 10x_3cl and 10x_5cl datasets, we conducted a contamination test [1, 42] and found that the additional cells called by Cell Ranger V3 introduced elevated contamination (Supplementary Fig. 6G). These findings indicated that inferCNV and CopyKAT may not be good options for low-quality data, as these methods might be sensitive to gene expression changes in individual cells, leading to false CNV identification. Overall, when used with a single platform, inferCNV, sciCNV, and CopyKAT performed well to identify cell subpopulations.

#### Subpopulation inference based on combined datasets from multiple scRNA-seq platforms

**Figure 5.**
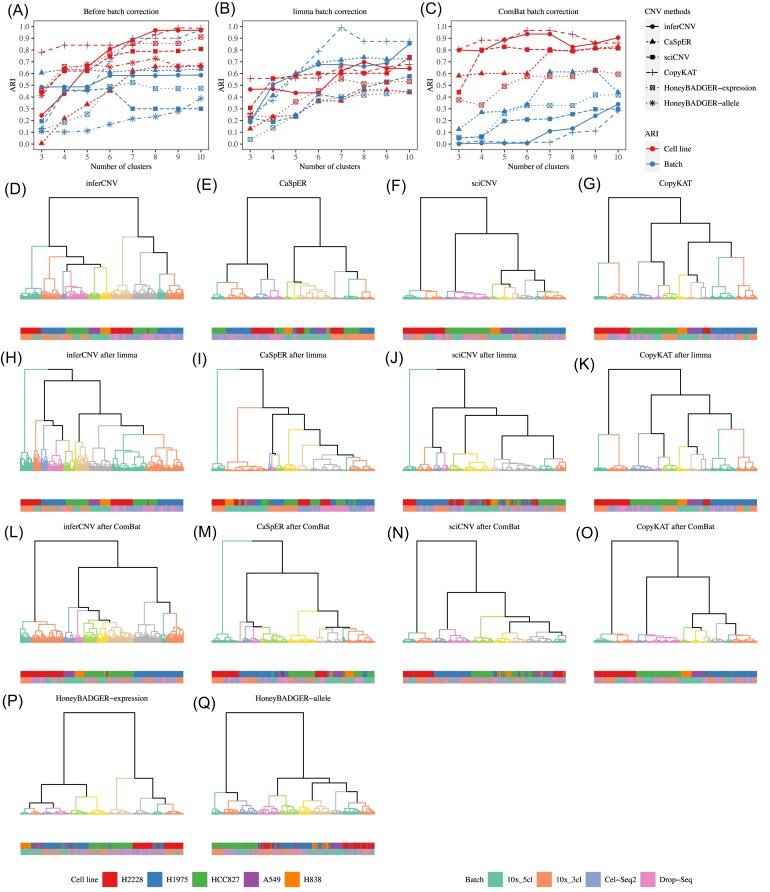
Accuracy of subpopulation identification based on scRNA-seq data from multiple platforms. (**A-C**) The ARI scores between true cell types or batches and the clusters estimated by the five CNV inference methods applied to the dataset (**A**) before, and (**B**) after limma, or (**C**) after ComBat batch corrections. (**D-Q**) Hierarchical clustering of the five CNV inference methods applied to the dataset before and after batch correction. The bottom bars represent the true cell line labels and batch labels. The HoneyBADGER scCNV method was evaluated separately based on either expression or allele.

Batch effects are a common issue in scRNA-seq analysis [30]. We simulated a test dataset with strong batch effects by combining datasets from different platforms. To create the combined dataset, we included all cells in the Drop-seq_3cl and CEL-seq2_3cl datasets and randomly selected 250 cells from each of the 10x_3cl and 10x_5cl (processed by Cell Ranger V2) datasets, resulting in a combined dataset consisting of 943 cells. In this scenario, we tested the five scCNV inference methods on the combined dataset and evaluated batch-based metrics. Well-performing methods exhibited a strong association between the clusters identified and the true cell lines, but a weak association with batch (defined by different scRNA-seq datasets or platforms). The HoneyBAGDER methods were less affected by batch effects, especially for the allele-based method, which achieved the best balance between cell line and batch based metric score when the number of clusters was five (Fig. 5A, P, Q). On the contrary, inferCNV, CaSpER, sciCNV, and CopyKAT were highly affected by batch effects (Fig. 5A, D-G, Supplementary Fig. 5A, D, G). To improve their performance, we applied batch correction methods before CNV inference. We observed increased cell line-based metric scores, and decreased batch based metric scores for all four scCNV inference methods (especially inferCNV and CopyKAT) after applying ComBat batch correction (Fig. 5I, O). Therefore, for subpopulation inference in the scRNA-seq data combined from multiple scRNA-seq datasets or platforms, inferCNV or CopyKAT combined with ComBat batch correction were the recommended options for inferring cell subpopulations. However, it should be noted that the batch corrected gene expression data may affect the accuracy of CNV detection. If the batch correction methods were not used, the HoneyBADGER methods, especially the allele-based HoneyBADGER, performed the best.

#### Rare cell subpopulation identification from the mixed lung cancer cell scRNA-seq datasets

In this scenario, we tested the sensitivity of five methods in identifying rare cell subpopulations using three scRNA-seq datasets from the mixed lung cancer cell lines: Drop-seq_3cl, CEL-seq2_3cl, and 10x_5cl [31]. To simulate a rare cell subpopulation, we selected a low percentage of cells from one specific cell line for evaluation [31]. The experimental design details are provided in Supplementary Table 2. In summary, for Drop-seq and CEL-seq2, we randomly selected a total of 100 cells from the three cell lines, with H2228 cells representing the rare cell subpopulation at low percentages (1%, 2%, 5%, and 10%). For 10x_5cl, we considered H1975 cells as the rare cell subpopulation and expanded the design to consider a total of 200, 500, and 1000 randomly selected cells. HoneyBADGER was not evaluated on 10x_5cl data due to its poor performance on that dataset.

Since the scCNV inference methods used Hidden Markov Models (HMM) to infer CNVs, there might have been a slight variation in the CNVs generated between different runs, which could influence cluster accuracy. To account for this, we repeated each scCNV inference method 10 times for the same dataset. We used three metrics to evaluate the performance: percentage of successful runs, number of clusters required to identify the rare cell subpopulation, and proportion of cells labeled as the rare cell subpopulation (see “Methods”)

The results of the evaluation are presented in Supplementary Table 3. Overall, inferCNV performed the best. CopyKAT and CaSpER performed similarly, though CopyKAT slightly outperformed CaSpER in most cases. sciCNV and HoneyBADGER are not recommended for identifying rare cell subpopulations.

We then focused primarily on evaluating inferCNV. For Drop-seq and CEL-seq2, at least 5% of cells were required for the rare subpopulation to be identified. For 10x, when the total number of cells was small (100 or 200 cells), inferCNV needed at least 5% of cells to be in the rare population to identify it. However, as the total number of cells increased, inferCNV exhibited increased sensitivity and it could identify the rare subpopulation cells even at 1% when the total number of cells exceeded 500. A similar trend was observed for other scCNV methods as well, indicating that the sensitivity of the scCNV inference methods in identifying rare cell subpopulations depends on both the percentage and the exact number of rare cells. For inferCNV, if the total number of cells exceeded 500, rare cells could be detected when present at least 1%. If the total number of cells decreased to 200 or lower, a percentage of 5% or higher was required for accurate identification.

### CNV detection using scRNA-seq data derived from a clinical SCLC study.

**Figure 6.**
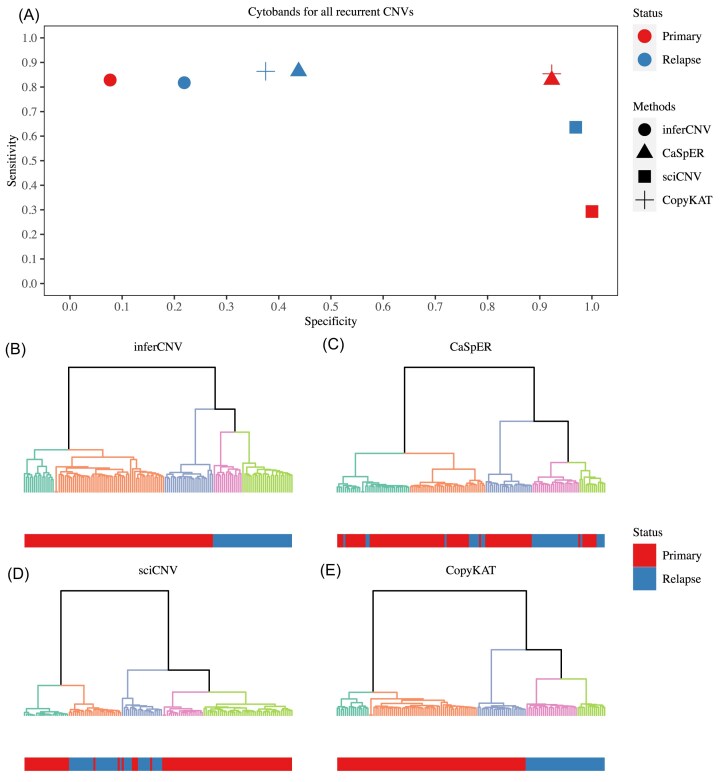
Evaluation of scCNV inference methods using a clinical small cell lung cancer (SCLC) dataset. (**A**) Sensitivity and specificity of the four scCNV inference methods applied to primary and relapse SCLC datasets. (**B-E**) Hierarchical clustering of the four scCNV inference methods applied to the SCLC dataset.

To further validate our findings, we compared CNVs detected by four methods (excluding HoneyBADGER) using a clinical dataset of human small cell lung cancer (SCLC). Similarly, we used CNVs identified by subHMM in WGS data from primary and relapse samples of the same patient as the ground truth. For the comparison of cytoband-based CNVs, we considered 54 highly recurrent CNVs (denoted as cytobands) from a reference paper [7, 38] as the total events. These cytobands were divided into 27 CNV gains and 27 CNV losses. In the primary scRNA-seq SCLC data, we observed results similar to our evaluation (Fig. 6A); CaSpER and CopyKAT exhibited the best performance in terms of sensitivity and specificity. However, in the relapse data, these two scCNV inference methods showed low specificity. This discrepancy might be attributed to the selection of the 54 highly recurrent CNVs, which were detected mainly in primary SCLC samples. In the evaluation of subpopulations, the results were consistent with the conclusions drawn above. inferCNV and CopyKAT outperformed other methods and were able to cluster primary and relapse cells with high accuracy (Fig. 6B-E).

## Discussion

In this study, we evaluated the capability of five scCNV inference methods across 8 datasets representing different scRNA-seq platforms including both tag-based and full-length transcript scRNA-seq technologies [30, 43]. We also applied the methods to a clinical dataset for validation. Two major aspects were considered in the evaluation, accuracy (sensitivity and specificity) of inferred CNVs and accuracy of subclones identified. To evaluate the accuracy of inferred CNVs, the CNVs identified by WGS data were used as ground truth [7, 34, 35]. In the evaluation, four different factors were considered: number of cells, normal reference samples, read length, and sequencing depth. We observed large variations of CNVs inferred by the five scCNV inference methods, resulting in quite different sensitivity and specificity (Fig. 2A-C). HoneyBADGER was the most conservative method; it identified the fewest CNVs and had the lowest sensitivity and highest specificity. inferCNV and sciCNV showed opposite patterns in terms of sensitivity and specificity, indicating either high false negative or low true positive rate. CaSpER and CopyKAT achieved the best balance between sensitivity and specificity.

The reference dataset affected scCNV inference results. Overall, the best performance was achieved using scRNA-seq reference data (Fig. 3A). When the two bulk RNA-seq reference datasets were used, CopyKAT outperformed other methods (Fig. 3A-C). Furthermore, we confirmed that CopyKAT worked extremely well for tag-based scRNA-seq platforms, especially the 10x platform, with all three normal reference datasets (Fig. 3C). We speculate that this was because CopyKAT was originally trained on 10x scRNA-seq data during development, and over 90% of our testing data were from this platform. Also, we found that CaSpER was not robust with different read depths/lengths since the method requires BAF signal profiles, which may vary significantly with different read depths/lengths.

We found it interesting that CaSpER led to inconsistent sensitivity and specificity at different read depths of the same sample (Fig. 3G, Supplementary Fig. 3). We believe the inconsistency was due to a large variation in BAF shift thresholds estimated from different BAF signal profiles at each read depth. For example, in the ICELL8 125bp case, we found the highest BAF shift thresholds at a read depth of 250K as compared with the other four read depths (Supplementary Table 4). In the CaSpER algorithm, the estimation of BAF shift threshold was critical for CNV filtering. The high BAF shift threshold resulted in a large number of filtered CNVs and low sensitivity. In our analysis, we used only the recommended parameter settings to extract BAF. A better fine-tuning of parameters in the current BAF extracting algorithm or developing a better BAF extracting algorithm might improve CaSpER analysis substantially.

Batch effects pose a significant challenge in scRNA-seq datasets, and it is critical to perform a batch effect correction to the scRNA-seq data using appropriate methods, as thoroughly evaluated in our previous study [30]. Most batch effect correction or integration methods for scRNA-seq focus on grouping the same cell types from different batches to enhance cell type clustering. However, these methods typically do not modify the original gene expression data but rather produce low-dimensional embeddings for clustering purposes. This approach is not suitable for CNV inference, which relies on the raw gene expression data for CNV detection. Currently, the primary option for correcting batch effects in CNV inference is to apply tools like ComBat or limma. For newly developed scRNA-seq CNV inference methods, it is essential to account for batch effects and incorporate them as a factor within the model to ensure accurate CNV calling.

In terms of subpopulation identification, inferCNV and CopyKAT showed better performance with scRNA-seq data from a single platform than with data derived from multiple platforms using the mixed samples [31]. However, with multiple platforms, all methods except HoneyBADGER were deeply affected by batch effects [30]. We tested two batch correction methods, limma [44] and ComBat [45], which were designed for bulk RNA-seq, and found ComBat improved the performance of inferCNV and CopyKAT dramatically. The recently developed scRNA-seq batch correction methods seemed not to work well with current scRNA-seq CNV inference methods because most of them only provided embedding matrices or normalized gene expression matrices instead of batch corrected gene expression matrices [30]. Since batch effects strongly affected the scRNA-seq CNV inference methods, we believe it is essential to develop novel scRNA-seq batch correction methods that overcome this limitation.

Computational efficiency is critical for single cell analysis because current scRNA-seq techniques can generate gene expression data from a few hundred to hundreds of thousands of single cells. In our study, we tested the dataset used in the “multiple platform inference” section with a total of 943 cells and further down sampled to 100, 200, and 500 cells. Since inferCNV supports multicore running, we evaluated different numbers of cores (1, 5, 10, and 20) to run inferCNV. All runs were conducted on a server with 2x Intel Xeon E7-8860 CPUs at 2.2 GHz, 384 GB memory. CopyKAT required the least computational time. HoneyBADGER-allele based method requires an excessively long time (> 24 h) when 500 or more cells were analyzed (Supplementary Fig. 7). CaSpER and HoneyBADGER-expression based methods required less time compared with inferCNV with less than 20 cores. inferCNV benefited a lot from multicore running, especially when the number of cells increased. With 943 cells, the three methods CaSpER, HoneyBADGER-expression, and inferCNV (with 20 cores) had similar computational time (∼ 2 h).

## Conclusions

We observed large differences in performance between the five scCNV inference methods evaluated. CaSpER and CopyKAT showed better sensitivity and specificity, while inferCNV and CopyKAT were the best for identifying tumor subpopulations using scRNA data derived from a single platform compared with the scRNA-seq data from multiple platforms derived from a mixture of scRNA-seq datasets [31]. However, the expression based scCNV inference methods such as inferCNV, CaSpER, sciCNV, and CopyKAT were highly affected by batch effects when estimating tumor subpopulations in mixed samples using multiple platforms. In the clinical application, CaSpER and CopyKAT outperformed other methods in terms of sensitivity and specificity. Regarding subpopulation identification, inferCNV and CopyKAT achieved better performance. The overall performance and our recommendations on selecting scCNV method inference methods using scRNA-seq data were presented in Supplementary Table 5. Our findings address the limitations of the current methods and provide guidance for developing new ones.

## Supplementary Material

pbaf011_Supplemental_Files
