# Supplementary material for: A benchmarking study of copy number variation inference methods using single-cell RNA-sequencing data
Source: Precis Clin Med. 2025 Jun 4;8(2):pbaf011. doi: 10.1093/pcmedi/pbaf011 (PMC12204187; doi:10.1093/pcmedi/pbaf011)
Supplement: pbaf011_Supplemental_Files [file pbaf011_supplemental_files.zip › Suppl_figures_corrected.pdf]

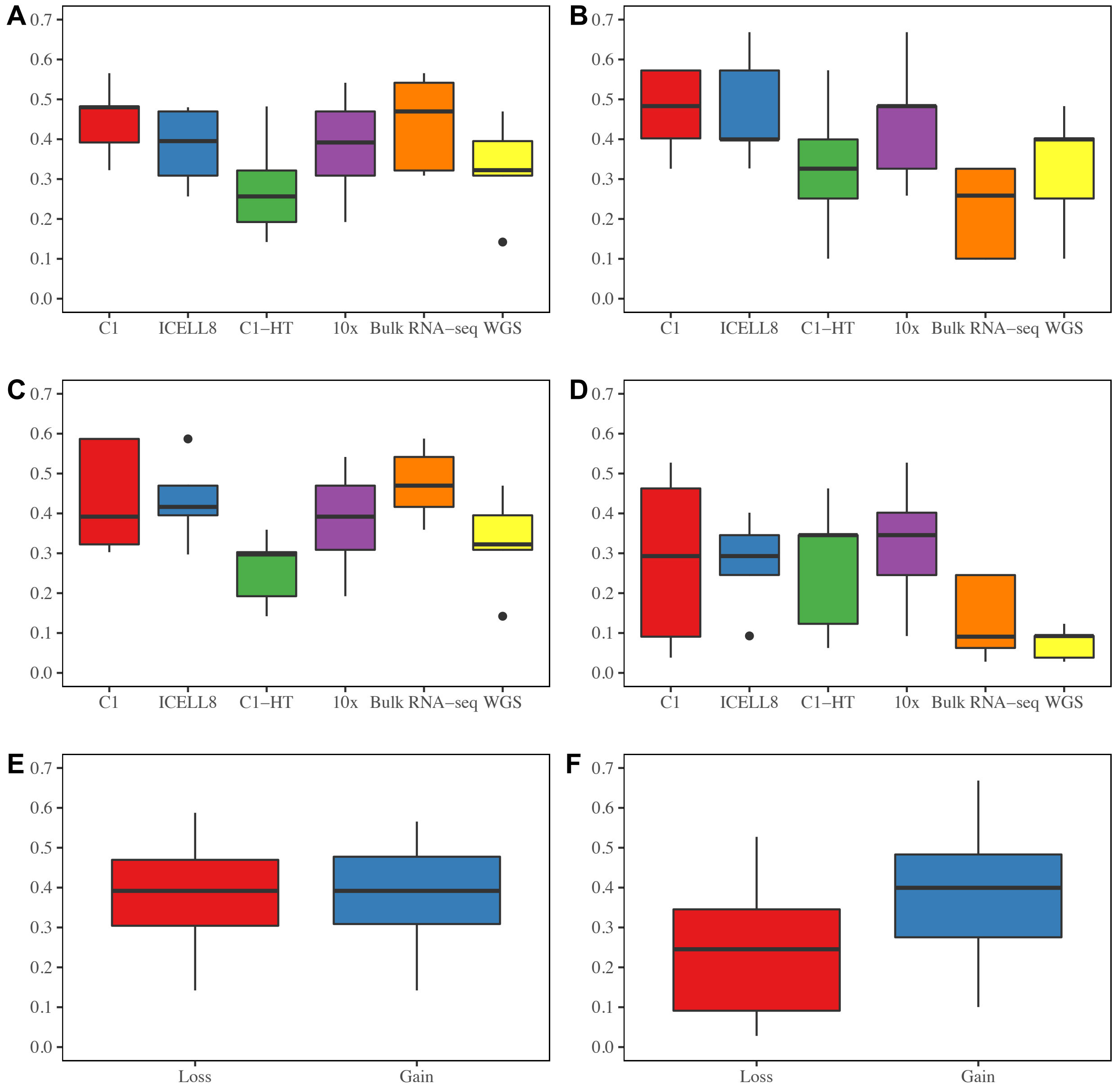

Supplementary Figure 1. Consensus of identified CNV (**A-B**) gains and (**C-D**) losses between any two protocols by (**A,C,E**) CaSpER and (**B,D,F**) CopyKAT. Each box represents the ARI between CNV identification status of the x-axis labeled protocol and other protocols.

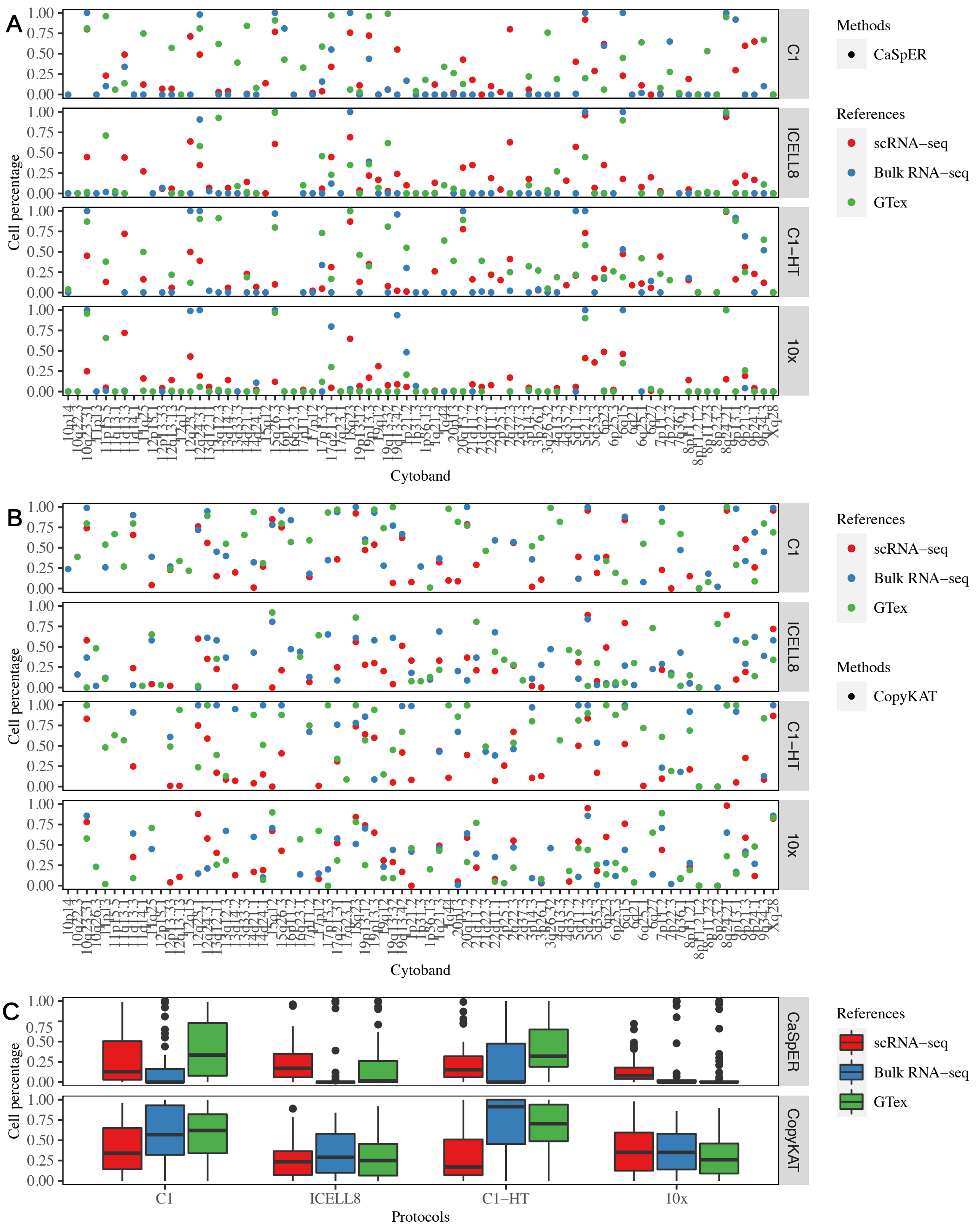

Supplementary Figure 2. Detection sensitivity of CaSpER and CopyKAT by using three normal references. (**A-B**) 79 highly recurrent CNVs that can be identified by (**A**) CaSpER and (**B**) CopyKAT at single cell level. Each dot represents the percentage of cells harboring the cytoband-based CNV with a specific protocol and a normal reference. (**C**) Boxplot showing the percentage of cells harboring the CNVs in CaSpER and CopyKAT.

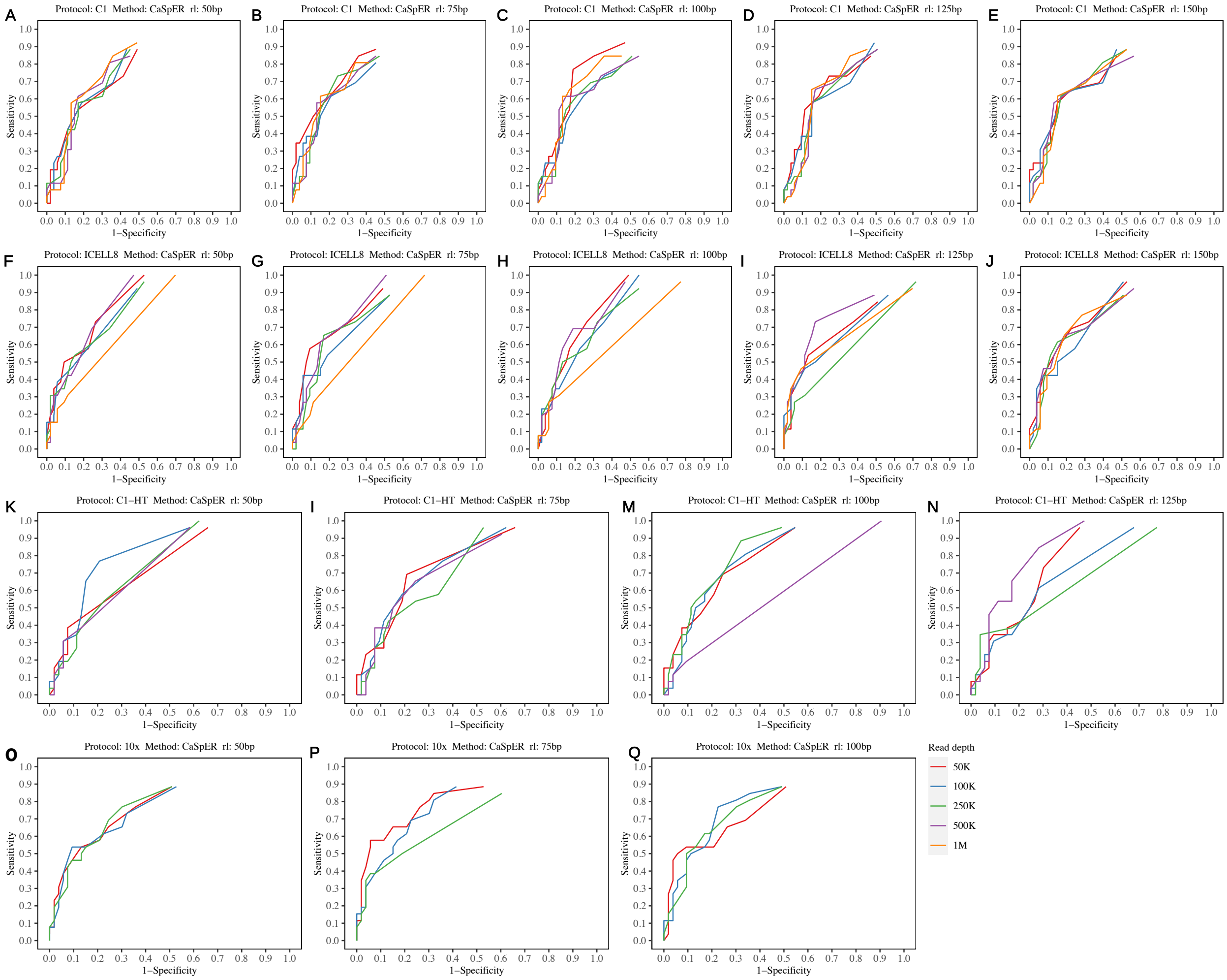

Supplementary Figure 3. ROC curves of CaSpER applied to four scRNA-seq protocols. The reads of each protocol were trimmed to specific read lengths and down sampled to specific read depths.

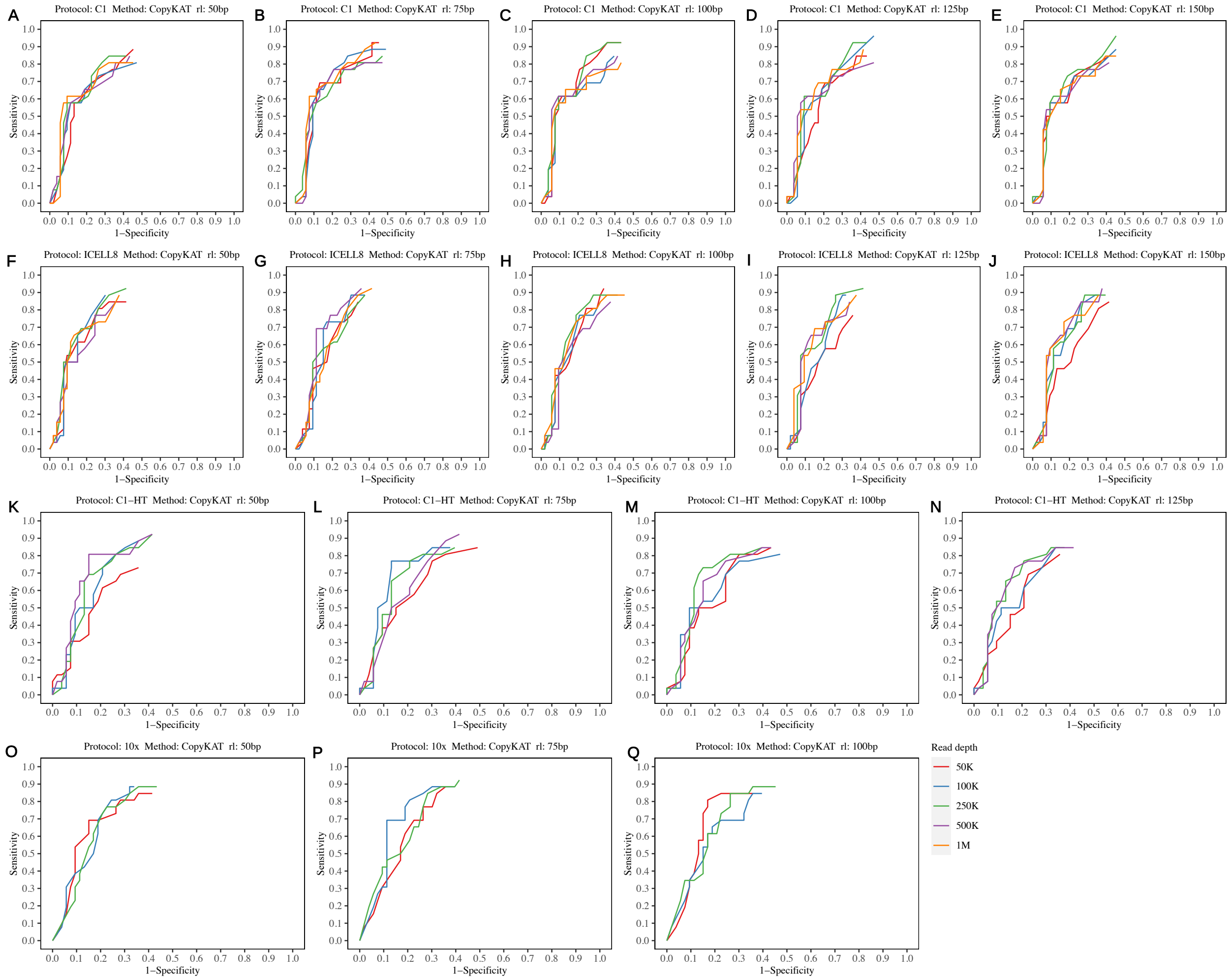

Supplementary Figure 4. ROC curves of CopyKAT applied to four scRNA-seq protocols. The reads of each protocol were trimmed to specific read lengths and down sampled to specific read depths.

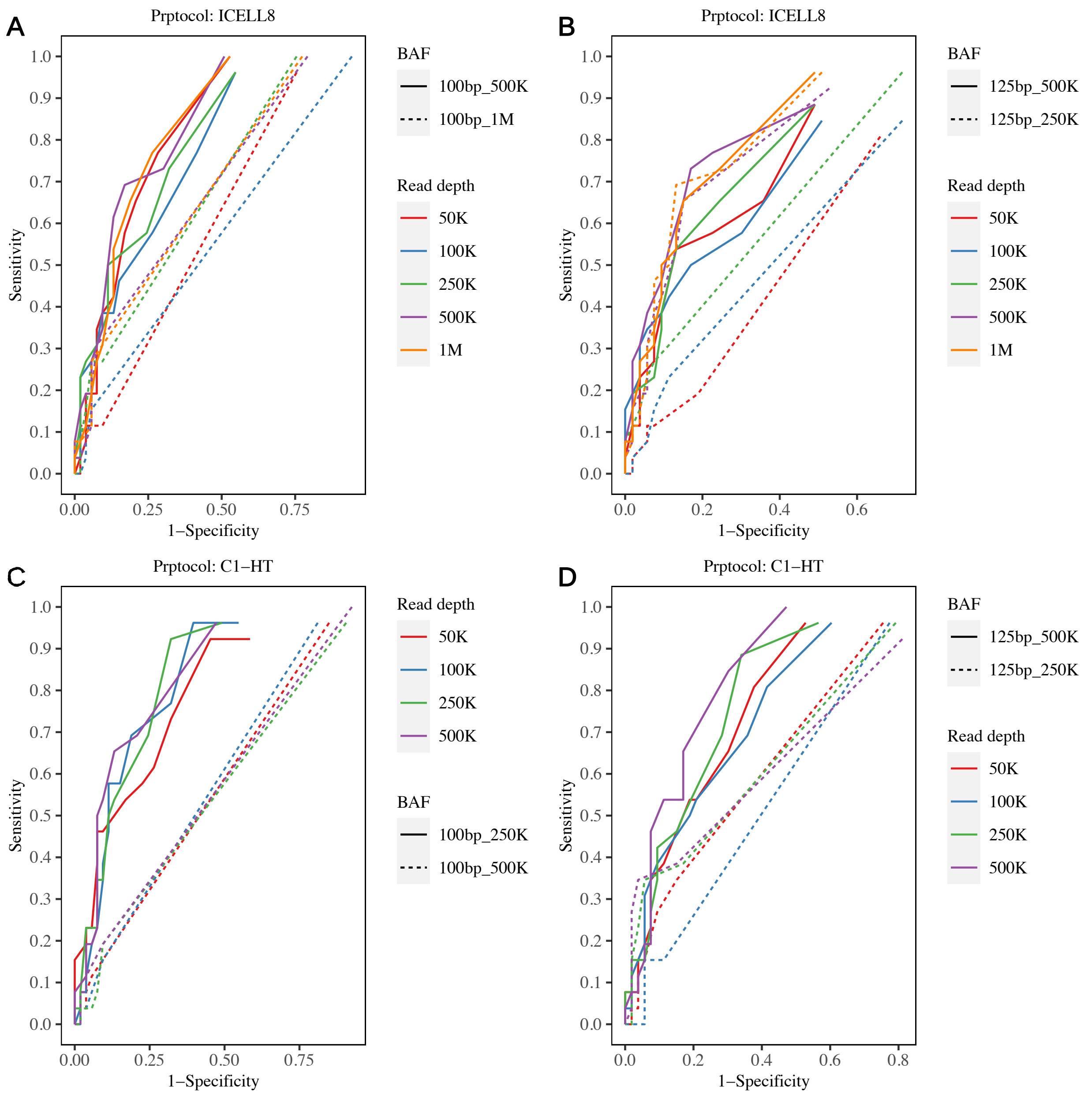

Supplementary Figure 5. Effect of BAF in CaSpER analysis. ROC curves of CaSpER applied to (**A-B**) ICELL8 and (**C-D**) C1-HT protocols using two BAF files.

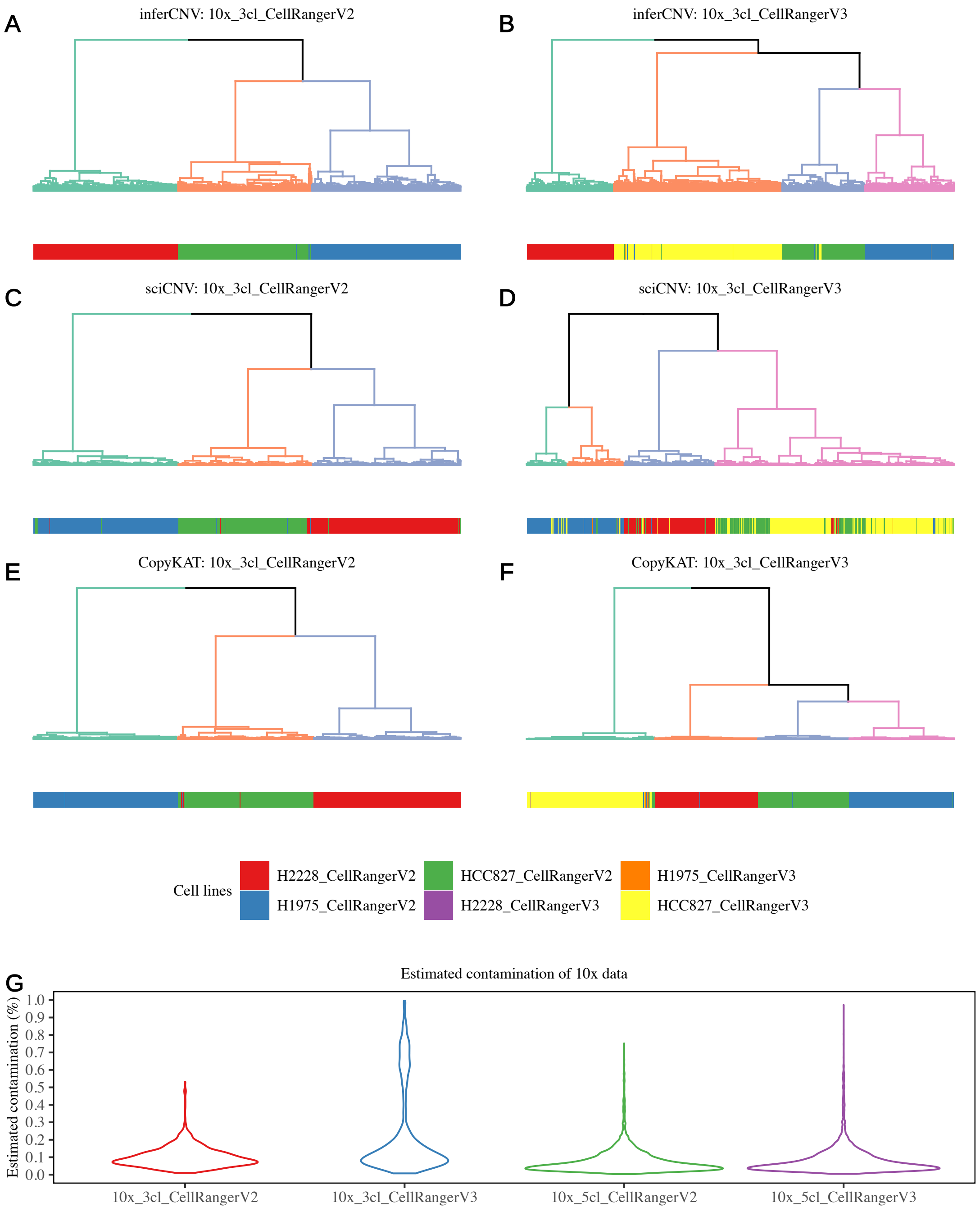

Supplementary Figure 6. Effect of low RNA content cells called by Cell Ranger V3. (A-**F**) Hierarchical clustering of the three CNV inference methods applied to the 10x_3cl data processed with (A,**C**,E) Cell Ranger V2 and (**B**,**D**,**F**) Cell Ranger V3, respectively. Cell lines label s as Cell Range V3 represent extra cells called by Cell Ranger V3. (**G**) Ambient RNA contamination levels of four 10x datasets estimated by DecontX.

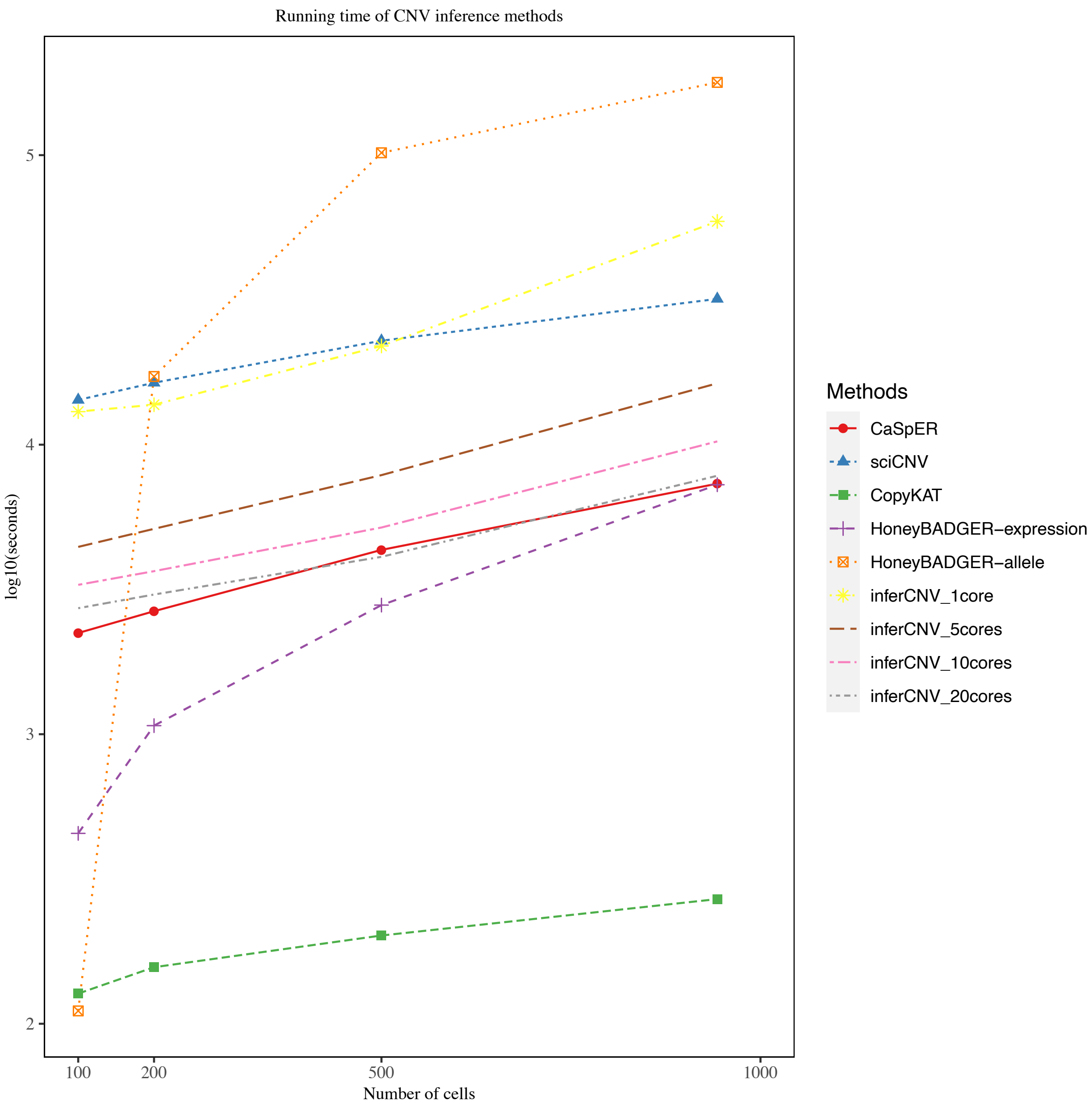

Supplementary Figure 7. Computational time (log10 transform) of the five CNV inference methods applied for four datasets with different number of cells.
