# Supplementary material for: A benchmarking study of copy number variation inference methods using single-cell RNA-sequencing data
Source: Precis Clin Med. 2025 Jun 4;8(2):pbaf011. doi: 10.1093/pcmedi/pbaf011 (PMC12204187; doi:10.1093/pcmedi/pbaf011)
Supplement: pbaf011_Supplemental_Files [file pbaf011_supplemental_files.zip › Supplementary_Table_2._docx.docx]

| **Supplementary Table 2**. Composition of cells in three mixed scRNA-seq datasets designed for rare subclones evaluation | | | | | | |
| --- | --- | --- | --- | --- | --- | --- |
| Design* | 10x_5cl | | | Drop-seq_3cl and CEL-seq2_3cl | | |
|  | A549 | H2228 | H1975 | H1975 | HCC827 | H2228 |
| 99_1 | 50 | 49 | 1 | 50 | 49 | 1 |
| 98_2 | 49 | 49 | 2 | 49 | 49 | 2 |
| 95_5 | 48 | 47 | 5 | 48 | 47 | 5 |
| 90_10 | 45 | 45 | 10 | 45 | 45 | 10 |
| 199_1 | 100 | 99 | 1 | NA | NA | NA |
| 198_2 | 99 | 99 | 1 | NA | NA | NA |
| 195_5 | 98 | 97 | 5 | NA | NA | NA |
| 190_10 | 95 | 95 | 10 | NA | NA | NA |
| 499_1 | 250 | 249 | 1 | NA | NA | NA |
| 498_2 | 249 | 249 | 2 | NA | NA | NA |
| 495_5 | 248 | 247 | 5 | NA | NA | NA |
| 490_10 | 245 | 245 | 10 | NA | NA | NA |
| 999_1 | 500 | 499 | 1 | NA | NA | NA |
| 998_2 | 499 | 499 | 1 | NA | NA | NA |
| 995_5 | 498 | 497 | 5 | NA | NA | NA |
| 990_10 | 495 | 495 | 10 | NA | NA | NA |
| *The first and second number in the design column represent the number of cells in major and rare subclones. | | | | | | |
